# Anomalous origin of the left coronary artery from the pulmonary artery (ALCAPA) presenting with ventricular fibrillation in an adult: a case report

**DOI:** 10.1186/1749-8090-3-33

**Published:** 2008-05-26

**Authors:** Thomas Kristensen, Klaus Fuglsang Kofoed, Steffen Helqvist, Morten Helvind, Lars Søndergaard

**Affiliations:** 1 Department of Radiology, Diagnostic Centre, Rigshospitalet, University of Copenhagen, Denmark; 2 Department of Cardiology and Thoracic Surgery, The Heart Centre, Rigshospitalet, University of Copenhagen, Denmark

## Abstract

Anomalous origin of the left coronary artery from the pulmonary artery (ALCAPA) is a rare congenital anomaly. The usual clinical course is severe left sided heart failure and mitral valve insufficiency presenting during the first months of life. However, in some cases collateral blood supply from the right coronary artery is sufficient and symptoms may be subtle or even absent. Arrhythmias or sudden cardiac death in adult life may be the first clinical presentation in patients with ALCAPA. We report a case, where a 39-year old woman presented with ventricular fibrillation during phycial exertion. Coronary angiography and CT-angiography revealed an anomalous origin of the left coronary artery, and an aortic reimplantation of the left coronary artery was performed followed by ICD implantation. A review of the literature on ALCAPA is presented along with CT images before and after surgery.

## Background

In approximately one percent of the general population a coronar artery anomaly (CAA) is found [1,2]. A CAA may involve an abnormal number, origin and/or course, termination or structure of the coronary arteries. Most CAAs are discovered as incidental findings during coronary angiography and are clinically insignificant. However, some CAAs are associated with clincal manifestations such as sudden cardiac death, arrhythmias, myocardial ischemia (fixed or episodic) or congestive heart failure [3].

Anomalous origin of the left coronary artery from the pulmonary artery (ALCAPA) is a rare congenital anomaly occurring in 1 of 300.000 births. Anomalous origin of a coronary artery was first described in1882 by Brooks [4]. However, the first clinical description was not published until 1933 by Bland, White and Garland [5], and the condition is also known as Bland-White-Garland-syndrome.

In patients with ALCAPA the pulmonary vascular resistance and pulmonary arterial pressure decrease shortly after birth, along with oxygen content of the pulmonary artery. This causes a drop in antegrade flow and oxygen content of the anomalous left coronary artery, leading to myocardial ischemia. This may progress to myocardial infarction during periods of increased myocardial oxygen consumption. Collateral circulation between the right and left coronary systems ensues and left coronary artery flow reverses and enters the pulmonary trunk due to the low pulmonary arterial pressure (coronary steal phenomena). Consequently, the myocardium remains inadequately perfused (fixed ischemia).

The typical clinical course is severe left sided heart failure and significant mitral valve insufficiency, presenting at the age of one to two months, where the pulmonary vascular resistance drops. Initial symptoms are feeding difficulties, irritability, diaphoresis, tachypnoea and tachycardia. Chest pain due to myocardial ischemia could be mistaken with infantile colic.

However, in some cases collateral blood supply from the right coronary artery may be sufficient and the patient could pass through childhood with relatively minor symptoms. In adult life symptoms may range from dyspnoea, chest pain and exercise intolerance to sudden cardiac death due to acute ischemia during exercise or malignant ventricular arrhythmias generated from myocardial scar tissue [6,7].

## Case presentation

A 39-year old woman experienced a sudden loss of consciousness during coitus. Her husband found no pulse, initiated cardiopulmonary resuscitation and placed an emergency call. The mobile acute care team arrived 2 minutes after the alarm and found ventricular fibrillation. After defibrillation the patient converted to sinus rhythm and upon admittance to the hospital she was hemodynamically stable and in sinus rhythm with some premature ventricular beats. As the patient was somnolent hypothermia treatment was initiated and a subsequent cerebral CT scan was found to be normal. Serial coronary enzymes were negative and ECG was without ST-segment changes. Echocardiography showed hypokinesia of the left ventricular anterior wall with an ejection fraction of 35% and normal heart valvular function. After three days the patient was extubated and physical examination revealed no neurological deficits.

During childhood she had been followed due to mild impaired left ventricular function thought to be due to endocardial fibroelastosis. In adult life she had been asymptomatic and had four uneventful pregnancies.

**Figure 1:**
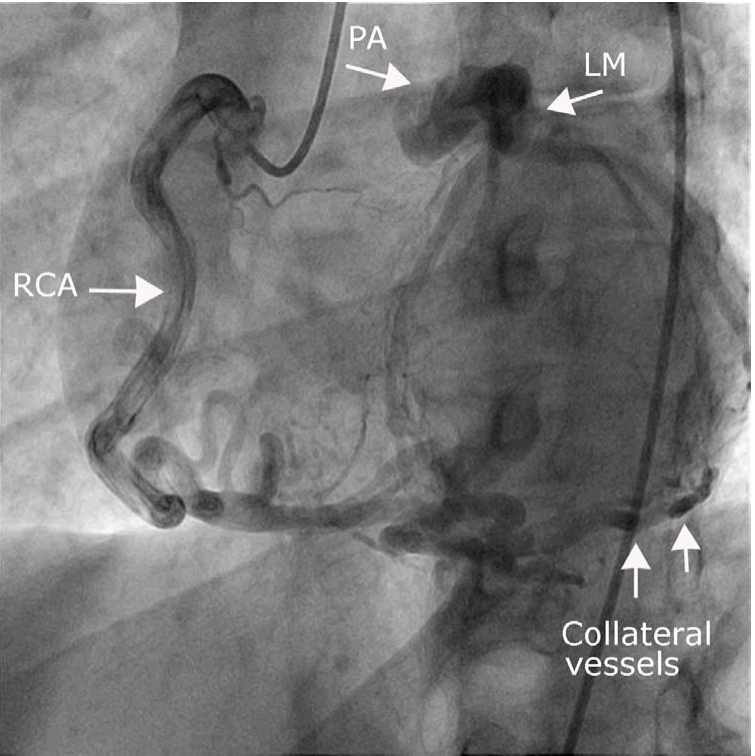
**Coronary angiography**. Coronary angiography of the right coronary artery with collateral filling of the left coronary vascular territory, which connects directly to the pulmonary trunk.

**Figure 2:**
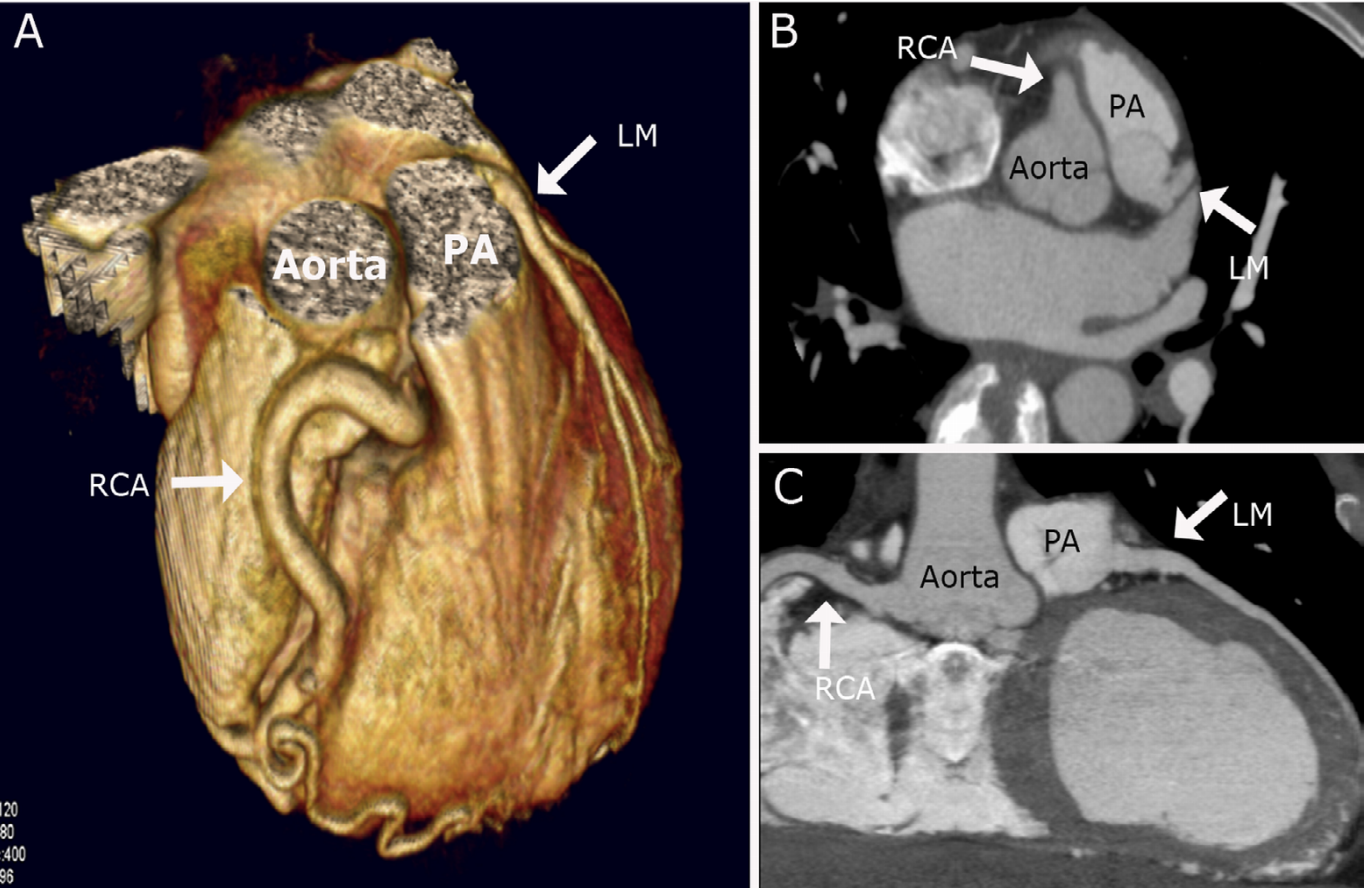
**CT-angiography before operation**. Coronary CT-angiography showing an anomalous origin of the left main coronary artery (LM) from the pulmonary artery (PA). Normal course of a very large right coronary artery (RCA) is seen. Volume-rendered reformation (A), axial (B) and curved multiplanar reconstruction (C).

**Figure 3:**
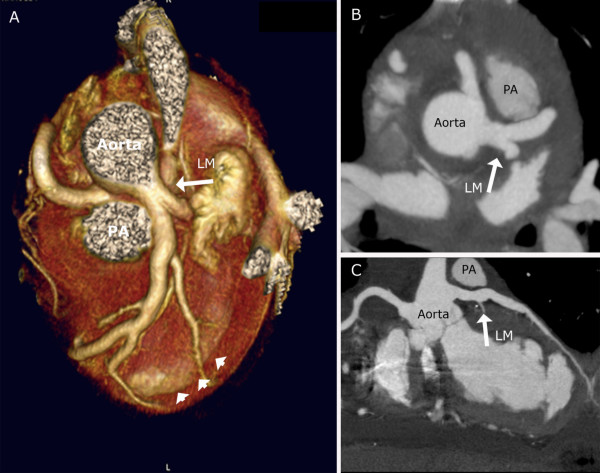
**CT-angiography after operation**. Coronary CT-angiography one week after aortic reimplantation of the left coronary artery confirms patency. Note the myocardial wall thinning in the apical anterior part of the left ventricle demonstrating the old infarction (arrowheads). Volume-rendered reformation (A), axial (B) and curved multiplanar reconstruction (C).

The patient was transferred to the cardiac catheterization laboratory for coronary angiography and further evaluation. Coronary angiography showed an anomalous left coronary artery arising from the pulmonary artery (ALCAPA) with retrograde filling through collaterals from an enlarged right coronary artery (figure 1). ECG-gated CT-angiography subsequently determined that a short distance between the ostium of left coronary artery and the aortic sinus would permit aortic reimplantation of the vessel (figure 2). Consequently, the patient underwent an uncomplicated aortic reimplantation of the left main coronary artery, and a CT-angiography one week after surgery confirmed the patency of the artery (figure 3). During surgery the presence of an old infarction in the anterior myocardial wall was noted. Based on the clinical history, with a documented episode of ventricular fibrillation (VF) without evidence of acute ischemia, a cardioverter defibrillator (ICD) was implanted.

## Discussion

85% of all cases of ALCAPA present within the first two months of life. However, symptoms may be misinterpreted (as in our case) or even be absent. In adult life patients with ALCAPA could present with symptoms of heart failure, mitral valve insufficiency, angina or arrhythmias. There are two potential substrates for arrhythmias in these patients. Firstly, areas with scar tissue after previous myocardial infarctions (MI) may alter the conduction in the left ventricle and become arrhythmogenic. Secondly, arrhythmias could be triggered from an acute ischemic event during exercise, where coronary steal phenomena may cause inadequate perfusion. Syncope due to ventricular tachycardia (VT) or cardiac arrest due to VF is therefore a major clinical presentation of ALCAPA in adults.

Objective findings include cardiomegaly on chest X-ray, and ECG may display an anterolateral infarct pattern. In children, the diagnosis can often be made by two-dimensional echocardiography with direct visualization of the abnormal origin of the left coronary artery and retrograde flow into the pulmonary artery. However, in adults the origins of the coronary arteries may be difficult to visualize, and in cases where the clinical suspicion is strong, a coronary angiography or CT-angiography should be performed.

In cases of ALCAPA several surgical treatment options have been proposed. Ligation of the anomalous artery at its origin in order to prevent coronary steal phenomena is one option, but this method depends on extensive collateral supply from the right coronary artery. An alternative percutaneous treatment approach was recently introduced by which the left coronary artery was closed by device embolization in a patient with large collaterals and coronary steal phenomena [8]. Today surgical procedures are aimed at creating a two-coronary system either via 1) a bypass graft (mammary artery or saphenous vein) in combination with ligation of the anomalous artery, 2) the Takeuchi-procedure where an intrapulmonary tunnel from the aortopulmonary window to the coronary artery is created or 3) translocation of the left coronary artery from the pulmonary trunk to the aortic sinus [9,10]. The latter depends on the distance between the origin of the anomalous artery and the aorta, but is possible in the majority of the cases [11].

In infants, most of the patients with corrected ALCAPA show normalization of both ventricular function and mitral valve insufficiency [12,13]. Estimated long-term survival at 20 years was recently shown to be 94.8% [13]. Simultaneous correction of the mitral valve insufficiency can be performed, but conservative treatment is often recommended as mitral valve function improves spontaneously.

No long-term studies of large populations of adults with corrected ALCAPA are available, but the prognosis is generally good. The late outcome after revascularization mainly depends on the extend of irreversible impaired left ventricular function and the presence of myocardial scar tissue.

Restoration of a dual coronary system will prevent further ischemia and arrhythmias of acute ischemic origin, but the anatomical substrate for ventricular arrhythmias in patients with old MI will not be altered after revascularization. As in other patients with a history of VT/VF not associated to an acute ischemic event, antiarrhythmic treatment must be considered. Treatment options include drug therapy, ICD implantation or catheter ablation. ICD implantation has been shown to be superior to drug treatment in patients with a history of VT/VF and previous MI (secondary prophylaxis) – especially when there is left ventricular dysfunction [14]. With recurrent VT refractory to these treatment options, direct surgical ablation or resection of the arrhythmogenic focus is an option

ICD implantation may also be considered in patients without a history of arrhythmias, when there is marked left ventricular dysfunction and electrophysiological study shows inducible VT (primary prophylaxis) [15].

In conclusion, the diagnosis of ALCAPA should be considered in adults without evidence of ischemic heart disease presenting with arrhythmias, left sided heart failure with or without mitral valve dysfunction, since an early diagnosis and surgical treatment generally results in an excellent prognosis.

## Consent

Written informed consent was obtained from the patient for publication of this case report and any accompanying images. A copy of the written consent is available for review by the Editor-in Chief of this journal

## Competing interests

The authors declare that they have no competing interests.

## Authors' contributions

All authors' read and approved the final manuscript.

TK analysed the CT-angiography and participated in the design and drafting of the manuscript

KFK analysed the CT-angiography and participated in the design and drafting of the manuscript

SH performed cornary angiography and participated in the design and drafting of the manuscript

MH performed the surgical procedure and participated in the design and drafting of the manuscript

LS was responsible for patient care and participated in the design and drafting of the manuscript
